# Oral recombinant *Lactobacillus* vaccine targeting the intestinal microfold cells and dendritic cells for delivering the core neutralizing epitope of porcine epidemic diarrhea virus

**DOI:** 10.1186/s12934-018-0861-7

**Published:** 2018-02-09

**Authors:** Sunting Ma, Li Wang, Xuewei Huang, Xiaona Wang, Su Chen, Wen Shi, Xinyuan Qiao, Yanping Jiang, Lijie Tang, Yigang Xu, Yijing Li

**Affiliations:** 1 College of Veterinary Medicine, Northeast Agricultural University, Mu Cai Street No. 59, Xiang Fang District, Harbin, People’s Republic of China; 2 Heilongjiang Key Laboratory for Animal Disease Control and Pharmaceutical Development, Harbin, People’s Republic of China

**Keywords:** *Lactobacillus*, Porcine epidemic diarrhea virus, Dendritic cell-targeting peptide, Microfold cell-targeting peptide, Oral immunization

## Abstract

**Background:**

Porcine epidemic diarrhea caused by porcine epidemic diarrhea virus (PEDV) has led to serious economic losses to the swine industry worldwide. In this study, an oral recombinant *Lactobacillus casei* vaccine against PEDV infection targeting the intestinal microfold (M) cells and dendritic cells (DCs) for delivering the core neutralizing epitope (COE) of PEDV spike protein was developed with M cell-targeting peptide (Col) and dendritic cell-targeting peptide (DCpep). The immunogenicity of the orally administered recombinant strains was evaluated.

**Results:**

After immunization, significantly higher levels of anti-PEDV specific IgG antibodies with PEDV neutralizing activity in the sera and mucosal sIgA antibodies in the tractus genitalis, intestinal mucus, and stools were detected in mice orally administered with the recombinant strain pPG-COE-Col-DCpep/L393, which expressed DCpep and Col targeting ligands fused with the PEDV COE antigen, compared to mice orally immunized with the recombinant strain pPG-COE/L393 without the DCpep and Col targeting ligands. Moreover, in response to restimulation with the PEDV COE antigen in vitro, a significant difference in splenocyte proliferation response and Th2-associated cytokine IL-4 level was observed in the group of mice orally immunized with pPG-COE-Col-DCpep/L393 (*p* < 0.05) compared to the groups of mice that received pPG-COE-Col/L393 and pPG-COE-DCpep/L393, respectively.

**Conclusions:**

The intestinal M cells- and DCs-targeting oral delivery of genetically engineered *Lactobacillus* expressing the COE antigen of PEDV can efficiently induce anti-PEDV mucosal, humoral, and cellular immune responses via oral administration, suggesting a promising vaccine strategy against PEDV infection.
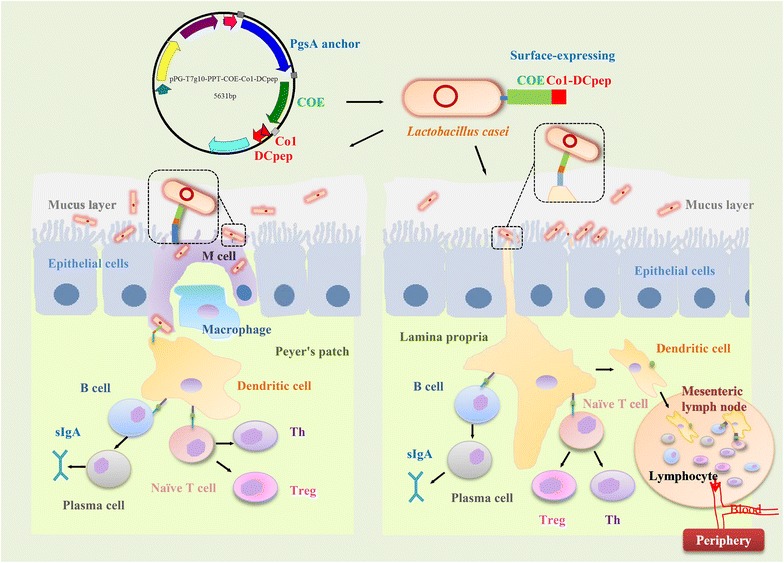

**Electronic supplementary material:**

The online version of this article (10.1186/s12934-018-0861-7) contains supplementary material, which is available to authorized users.

## Background

Porcine epidemic diarrhea (PED) characterized by vomiting, diarrhea, and dehydration, is a highly contagious intestinal disease caused by porcine epidemic diarrhea virus (PEDV). PEDV, a member of the *Coronaviridae* family, is an enveloped, single-stranded RNA virus whose genome comprises five open reading frames (ORFs) encoding an accessory protein and four structural proteins [1]. Of these, the spike on the surface of PEDV mediates binding and fusion between the virus and cells. In addition, the neutralizing epitope region of PEDV (COE) has been shown to induce virus neutralization antibodies and is used as a potential candidate immunogen against PEDV [2, 3]. PEDV has caused serious economic losses to the swine industry in Europe, America, Australia, and especially Asia. Currently, commercial vaccines, which mostly include inactivated vaccines [4] and attenuated PEDV vaccines, have provided some protection, but their efficacy has been poor [5, 6]. Since PEDV causes mainly intestinal infections [7], secretory IgA (sIgA), which can bind microbes and toxins in the intestine to prevent their adherence to epithelial cells [8], is desirable for defending the mucosa against PEDV. Thus, research on vaccination inducing a more efficacious sIgA-based protective mucosal immunity is urgently needed to prevent PEDV infections.

Oral mucosal vaccination has the great advantage of improving practicality for mass vaccination during pandemics, along with ease of production and administration. Above all, local protective mucosal immunity which is pivotal in response to PEDV invasion is generally not induced with parenteral vaccination [9]. Induction of mucosal immunity should be focused on the interaction between the antigen and lymphocytes within the mucosa. One promising approach involves the use of live recombinant *Lactobacillus* which can colonize the gastrointestinal tract and compete with pathogens for mucosal binding sites [10]. Cell wall associated or secreted factors from some *Lactobacillus* strains can enhance innate immune responses and epithelial barrier function, modulate the intestinal micro-environment, regulate immune cell behavior, and elicit release of cytokines [11]. In addition to natural immune-stimulating adjuvants with weak immunogenicity [12] and capacity to survive the gastric acid and digestive enzymes, several *Lactobacillus* strains have been applied for the delivery of heterologous antigens to trigger mucosal immune responses against pathogens [13–15].

Although a live recombinant *Lactobacillus* vaccine can propagate in the porcine intestine, only a small amount of antigen can reach the effective immune sites such as Peyer’s patches (PPs) [16]. To increase the bioavailability of the immunogen of interest, the addition of dendritic cell-targeting peptide (DCpep) and microfold cell (M cell)-targeting peptide (Col) to this oral *Lactobacillus* delivery system has been proposed. Microbial sampling commonly includes two principal mechanisms: (1) M cells, predominantly localized at PPs can transfer the antigen to DCs and macrophages for processing and (2) DCs pierce the epithelial layers to sample the antigen directly [17]. Previous studies have demonstrated that DCpep directs the protective antigen to DCs using *Lactobacillus* as a vector to induce the desired intestinal and systemic immune response [18–20]. Furthermore, M cell ligand-fused antigens have been shown to enhance the specific mucosal immune response [21, 22]. A combination of targeting to DCs and M cells simultaneously may further strengthen and improve the antigen uptake and presentation.

Therefore, in the present study, genetically engineered *Lactobacillus casei* 393 (*L. casei* 393, L393) strains, were constructed to constitutively express the COE of PEDV with either one or both of DCpep and Col on their cell surface. The immunogenicity of these recombinant strains was then investigated by oral administration in mice.

## Methods

### Virus, bacteria, plasmids, and cell line

PEDV HLJ-2012 was isolated from the intestines of piglets with severe diarrhea in Heilongjiang, China, and was maintained in our laboratory. *Lactobacillus casei* ATCC 393 was cultured in de Man Rogosa and Sharpe (MRS) broth at 37 °C. The constitutive expression plasmid pPG-T7g10-PPT, previously constructed by our laboratory, contained the HCE strong constitutive promoter, T7g10 transcriptional enhancer, PgsA anchor from *Bacillus subtilis* for stabilizing the heterologous protein in the cell membrane (surface-displaying), and the rrnBT1T2 terminator [23], and was used to construct *COE* plasmids as described below.

### Construction of recombinant *Lactobacillus* strains

**Fig. 1:**
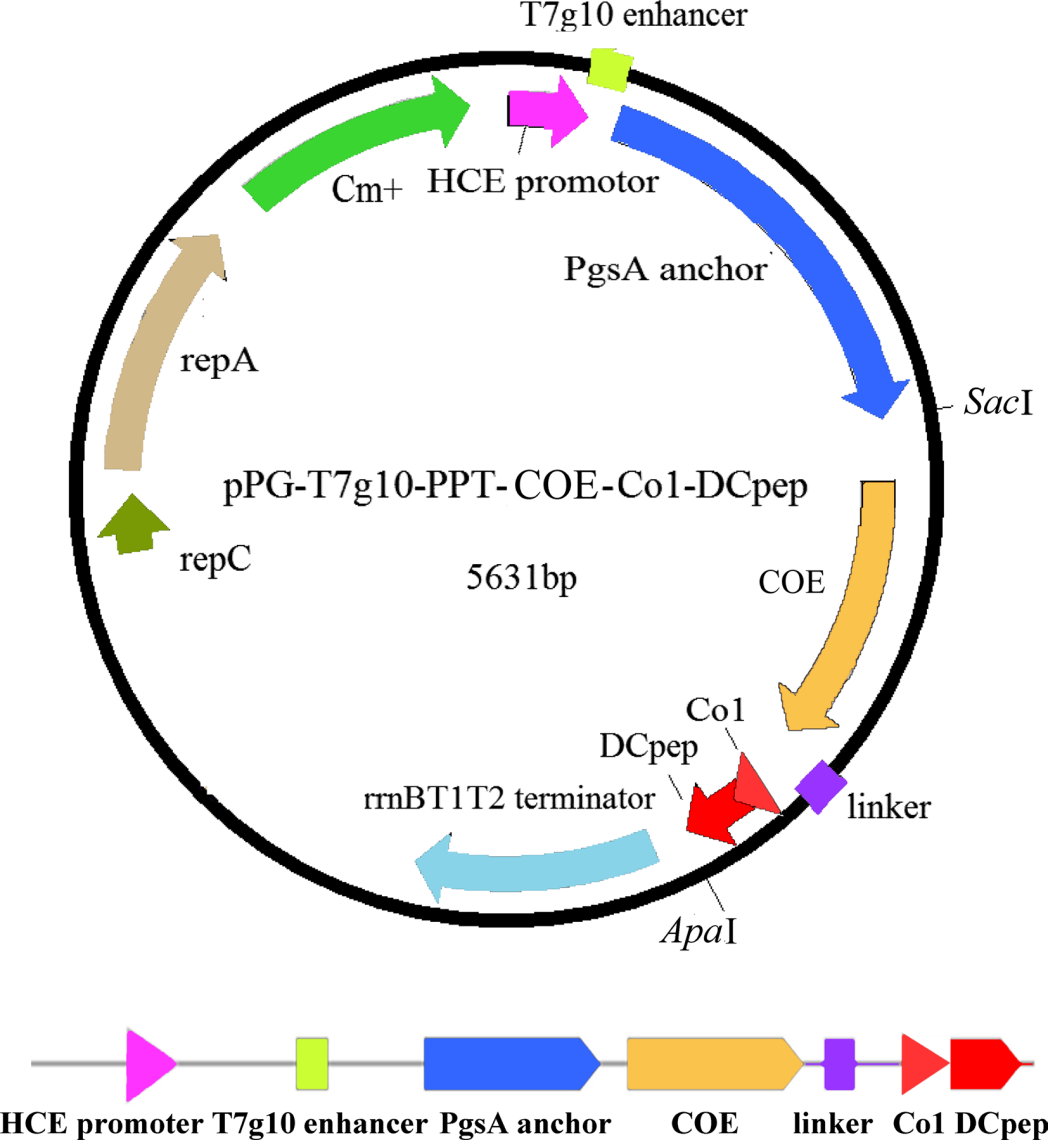
Construction diagram of recombinant plasmid pPG-COE-Col-DCpep. HCE strong constitutive promoter; T7g10 transcriptional enhancer; PgsA anchor from *Bacillus subtilis*; COE coding sequence; DC targeting peptide; M cell targeting peptide; rrnBT1T2 terminator; replicon C (rep C); replicon A (rep A); chloramphenicol coding sequence (Cm+)

A recombinant expression plasmid was constructed as shown in Fig. 1. Nucleic acid manipulations and cloning procedures were performed according to standard procedures. Total virus RNA was extracted using a commercial kit (Fastagen, Shanghai, China). First-strand cDNA was transcribed with Revert Aid M-MlV reverse transcriptase (Fermentas, New York, USA). Briefly, RNA was incubated with 2 μL (10 µmol L^−1^) of reverse primer for *COE* for 10 min at 75 °C. Subsequently, 1 mL of RNase Inhibitor (20 g mL^−1^), 2 mL of dNTP mix, and 1 mL of RevertAid M-MuLV reverse transcriptase were added, mixed gently, centrifuged at 1000×g, and incubated at 42 °C for 2 h. The reaction was terminated by heating at 70 °C for 5 min, and the cDNA template was used for polymerase chain reaction (PCR). Primers designed on the spike gene of PEDV HLJ-2012 (GenBank Accession No. JX512907.1) are listed in Table 1. Primers named COE-F1 and COE-R1 were used to obtain *COE* gene without targeting peptide. Then we use *COE* gene as PCR template, COE-F1 and COE-R2 as primers to get *COE*-*Col*-*DCpep*. The reverse primer named COE-R2 contained the sequence of targeting peptides to fuse *DCpep* and *Col* with the C terminus of *COE*. The flexible linker (GGGGS) was inserted between the two targeting peptides and flexible amino acids (GS) were inserted between *COE* and targeting peptide. SWISS-MODEL was used to predict the fused protein structure [24]. After that, the *COE*-*Col*-*DCpep* fusion genes were cloned into the expression plasmid pPG-T7g10-PPT, to generate pPG-T7g10-PPT-COE-Col-DCpep. To construct the recombinant lactobacillus strains, the recombinant plasmids were electrotransferred into *L. casei* 393 as described previously [25], giving rise to the recombinant strain pPG-COE-Col-DCpep/L393. In addition, pPG-T7g10-PPT-COE/L393, pPG-T7g10-PPT-*COE*-Col/L393 and pPG-T7g10-PPT-COE-DCpep/L393 were also generated and maintained in our laboratory.

**Table 1:** Sequence of primers

Primers	Sequence (5′–3′)
COE-F1	GAGCTCAAGCTTGTTACTTTGCCATCGTTT
COE-R1	GGGCCCTCAAACGTCCGTGACACCTTC
COE-R2	GGGCCCTTA**TGGACGTTGTGGAGTTGAATGATATGATGGATAAAA**^**a**^*TGAGCCACCGCCACC*^*b*^**TGGTAATGGTGAACGAGCTGGTAATTGATGAAATGA**^**c**^TGAACCTCTAGAAACGTCCGTGACACCTTC

Restriction enzyme recognition sites used for cloning are shown with underline

^a^ Dendritic cell-targeting peptide (DCpep) (in bold)

^b^ Flexible amino acid (in italic)

^c^ Microfold cell-targeting peptide (Col) (in bold)

### Analysis of protein expression by western blotting and immunofluorescence

Briefly, 2 mL of recombinant *L*. *casei* 393 was grown in MRS broth containing chloramphenicol (10 μg mL^−1^) at 37 °C for 12 h (OD_600_ ≈ 1), harvested by centrifugation, and washed twice with sterile PBS. After lysis of the sedimented cells with lysozyme, the lysate was incubated with sodium dodecyl sulfate (SDS) loading buffer in a boiling water bath for 10 min. The bacterial proteins were then separated by SDS-polyacrylamide gel electrophoresis (SDS-PAGE), and electrotransferred onto a polyvinylidene fluoride (PVDF) membrane. The immunoblots were probed with a mouse anti-COE serum (1:200) prepared in our laboratory as the primary antibody, and a horseradish peroxidase (HRP)-conjugated goat anti-mouse IgG (1:4000) as the secondary antibody.

Furthermore, for detection of the surface-displayed COE and targeting peptide of recombinant *L. casei*, immunofluorescence was used as described previously. In brief, recombinant strains were cultured overnight in MRS at 37 °C. The cell pellets were subsequently incubated with anti-PEDV COE serum (1:100) followed by fluorescein isothiocyanate-conjugated goat anti-mouse IgG (Invitrogen, USA) secondary antibodies (1:1000). Samples were then washed thrice with PBS, stained with 4,6-diamidino-2-phenylindole (DAPI) (Invitrogen, USA) for 30 min at 4 °C, washed three times, resuspended in 200 μL PBS, and then smeared on a microscope slide. Images were viewed by laser confocal microscopy (model LSM510 META; Zeiss, Germany).

### Immunization

Six-week-old female specific pathogen-free (SPF) BALB/c mice were purchased from Liaoning Changsheng Biotechnology Company in Liaoning, China, and kept under SPF conditions with free access to standard water and diet. All animal procedures were approved by the Ethical Committee for Animal Experiments (Northeast Agricultural University, Harbin, China). Prior to oral administration, the recombinant *Lactobacillus* strains were cultured overnight in MRS medium, washed with sterile PBS, and resuspended in PBS at a concentration of 10^10^ CFU mL^−1^. Sixty mice were randomly divided into six groups (10 mice per group): PBS, pPG/L393, pPG-COE/L393, pPG-COE-Col/L393, pPG-COE-DCpep/L393, and pPG -COE-Col-DCpep/L393 groups. The immunization dosage was 100 μL (10^9^ CFU) per mice, administered on 3 consecutive days (days 1, 2, and 3). Booster immunizations were administered on days 14, 15, and 16.

### Specimen collection

Serum and mucosal lavage samples from the external genital tract were obtained on days 0, 10, 17, 27, 34, 41 after immunization. Immunized mice (five mice in each group) were sacrificed to collect the intestinal tract (from duodenum to ileum) before the primary immunization. And five mice were sacrificed on day 40 after immunization to collect the intestinal tract and spleens, followed by washing with sterile PBS. Samples of the intestinal tract were stored at − 40 °C until analysis. In addition, fecal samples were collected on days 0, 4, 6, 8, 10, 12, 14, 16, 20, 22, 24, 26, 28, 30, 32, 34, and 36 after immunization, and treated according to a previously described method [26]. Briefly, 0.1 g of fecal pellets were suspended in 400 μL of PBS containing 1 mmol L^−1^ phenylmethylsulfonyl fluoride (Sigma, USA) and 1% bovine serum albumin (BSA), and then incubated at 4 °C for 16 h. After centrifugation, the supernatants were stored at − 40 °C until use.

### ELISA analysis of antibody levels

The levels of IgG in the sera and IgA in the external genital tract, intestinal tract, and stools were measured by ELISA. Polystyrene microtiter plates were coated overnight at 4 °C with PEDV NJ propagated on Vero cells and the culture of Vero cells used as a negative control for the antigen. After blocking with 5% skimmed milk, the collected samples were serially diluted in PBS, added in triplicate and incubated at 37 °C for 1 h. Then, an HRP-conjugated goat anti-mouse IgG or IgA antibody (Invitrogen, USA) was added to each well (1:5000) and incubated for 1 h at 37 °C. Color was then developed using o-phenylenediamine dihydrochloride (Sigma, USA) as a substrate, and absorbance at OD_490_ was measured.

### Proliferation of lymphocytes from immunized mice and cytokine ELISA

On day 40 post-immunization, splenocytes were obtained from immunized mice subjected to euthanasia for lymphocyte proliferation assay. In brief, 100 μL of the cell suspension (5 × 10^6^ cells mL^−1^), in eight duplicates, four of which were prepared for cytokine ELISA, were incubated in a 96-well plate containing RPMI 1640 medium plus 10% fetal calf serum at 37 °C in a 5% CO_2_ incubator. The cells were then restimulated with 5.0 μg mL^−1^ concanavalin A (ConA), 0.5 or 5.0 μg mL^−1^ of purified recombinant COE protein, and culture medium for 72 h. Control wells contained medium alone. Lymphocyte proliferation was assessed by a CellTiter 96 AQueous Non-Radioactive Cell Proliferation Assay according to the manufacturer’s instructions (Promega, USA), with absorbance measured at 570 nm. The stimulation index was calculated as follows: SI = OD_570: sample_/OD_570: blank control_.

Supernatant fluids (from the lymphocyte proliferation assay) were collected at 72 h, after which IL-4 and IFN-γ assays were performed using ELISA kits, according to the manufacturer’s instructions (Biosource International, USA). The cytokine concentrations were calculated according to the standard curve obtained for each ELISA plate.

### Neutralization assay

Briefly, sera from mice on day 41 post-immunization were filtered and inactivated at 56 °C for 30 min. The sera were serially diluted twofold (1:2, 1:4, 1:8, 1:16, 1:32, 1:64……), mixed with an equal volume of virus suspension (200 TCID50), and incubated at 37 °C for 1 h. The intermixture was inoculated onto a Vero cell monolayer at 37 °C in a 5% CO_2_ incubator. The positive serum control, negative serum control, virus control, and blank control were prearranged. Cytopathic effect (CPE) was observed daily. The Reed–Muench statistical method was used to calculate the results [27].

### Statistical analysis

Data are shown as the mean ± standard errors of three replicates per test in a single experiment repeated three times. Tukey’s multiple comparison tests were used to analyze differences between groups. A *p* value less than 0.05 was considered statistically significant, and a *p* value less than 0.01 was considered highly significant.

## Results

### Expression of the fusion protein

**Fig. 2:**
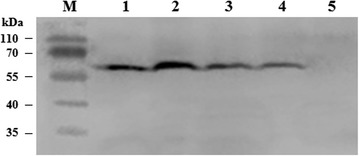
Expression of the fusion protein was identified by western blot. Mouse anti-COE serum was used as the primary antibody. M: Pre-stained protein marker; 1: pPG-COE/L393; 2: pPG-COE-Col/L393; 3: pPG-COE-DCpep/L393; 4: pPG-COE-Col-DCpep/L393; 5: pPG/L393. The calculated molecular mass of the fused COE and anchor is about 60 kDa

To ensure that the structure of the antigen and targeting peptides was not interfered, the structure of the fused protein was predicted. As shown in Additional file 1, DCpep and Co1 can expose at the C terminus of COE, and did not interfere with COE. To establish an oral vaccine delivery system, the constructed plasmids were successfully transformed into *Lactobacillus*. The recombinant *Lactobacillus* strains were cultured in MRS broth at 37 °C for 12 h, and the expression of COE was then determined by western blotting with a mouse anti-COE polyclonal antibody. As shown in Fig. 2, the fusion protein was about 60 kDa consistent with the predicted molecular weight. The negative control, pPG/L393, did not display a corresponding immunoreactive band.

**Fig. 3:**
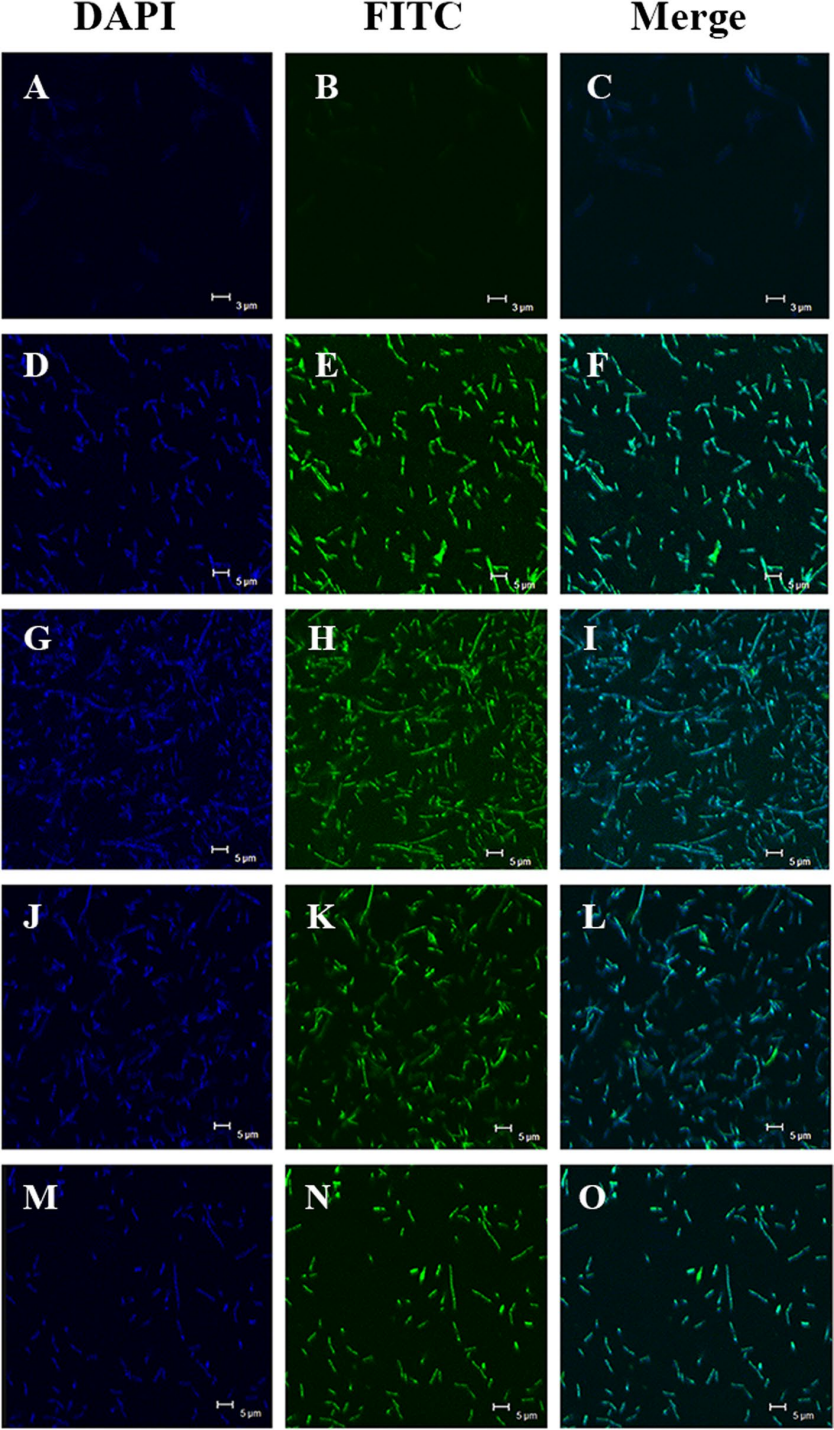
Heterologous protein displayed on the surface of *Lactobacillus casei*. Indirect immunofluorescence analysis was carried out according to standard procedures using mouse anti-COE serum and goat anti-mouse IgG conjugated with fluorescein isothiocyanate, as the primary and secondary antibody, respectively. **A**, **B**, **C** pPG/L393; **D**, **E**, **F** pPG-COE/L393; **G**, **H**, **I** pPG-COE-Col/L393; **J**, **K**, **L** pPG-COE- DCpep/L393; **M**, **N**, **O** pPG-COE-Col-DCpep/L393

Laser confocal microscopy was used to determine whether the fusion protein was expressed on the surface of the *Lactobacillus* cells. As shown in Fig. 3, after cells were incubated with FITC-conjugated secondary antibodies and stained with DAPI, pPG-COE/L393 (Fig. 3F), pPG-COE-Col/L393 (Fig. 3H), pPG-COE-DCpep/L393 (Fig. 3K) and pPG-COE-Col-DCpep/L393 (Fig. 3N) emitted green fluorescence on the cellular surface, whereas pPG/L393 did not (Fig. 3B), which indicates that the fusion protein was displayed on the surface of the bacteria.

### Immune responses induced in mice by oral administration of the recombinant strains

**Fig. 4:**
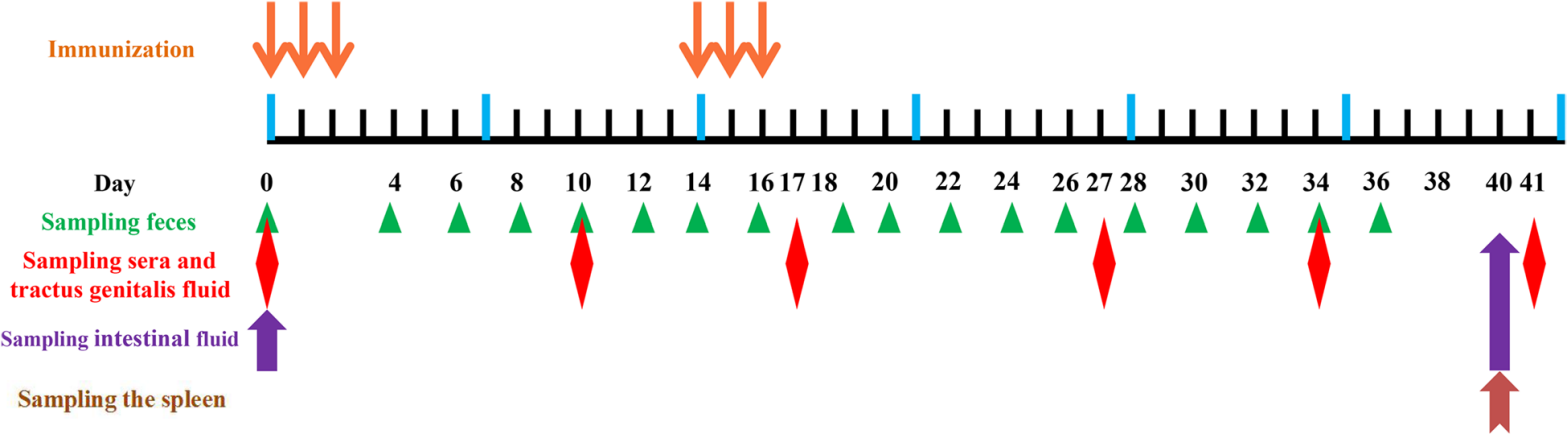
Scheme of oral immunization and schedule of sampling feces, sera, intestinal mucus, external genital tract fluids, and spleens. Immunization dosage was 100 μL (10^9^ CFU) per mice by oral administration. Primary immunizations were performed on 3 consecutive days (days 1, 2, and 3). Booster immunizations were administered on days 14, 15, and 16

**Fig. 5:**
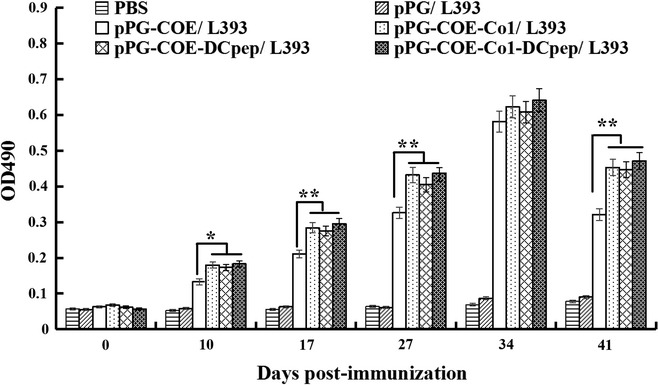
Anti-PEDV specific IgG antibody levels in the sera. Groups (n = 6) of BALB/c mice were orally immunized with recombinant strains and PBS. Bars represent the mean ± standard error value of each group (*p < 0.05, **p < 0.01 compared with pPG-COE/L393 group)

**Fig. 6:**
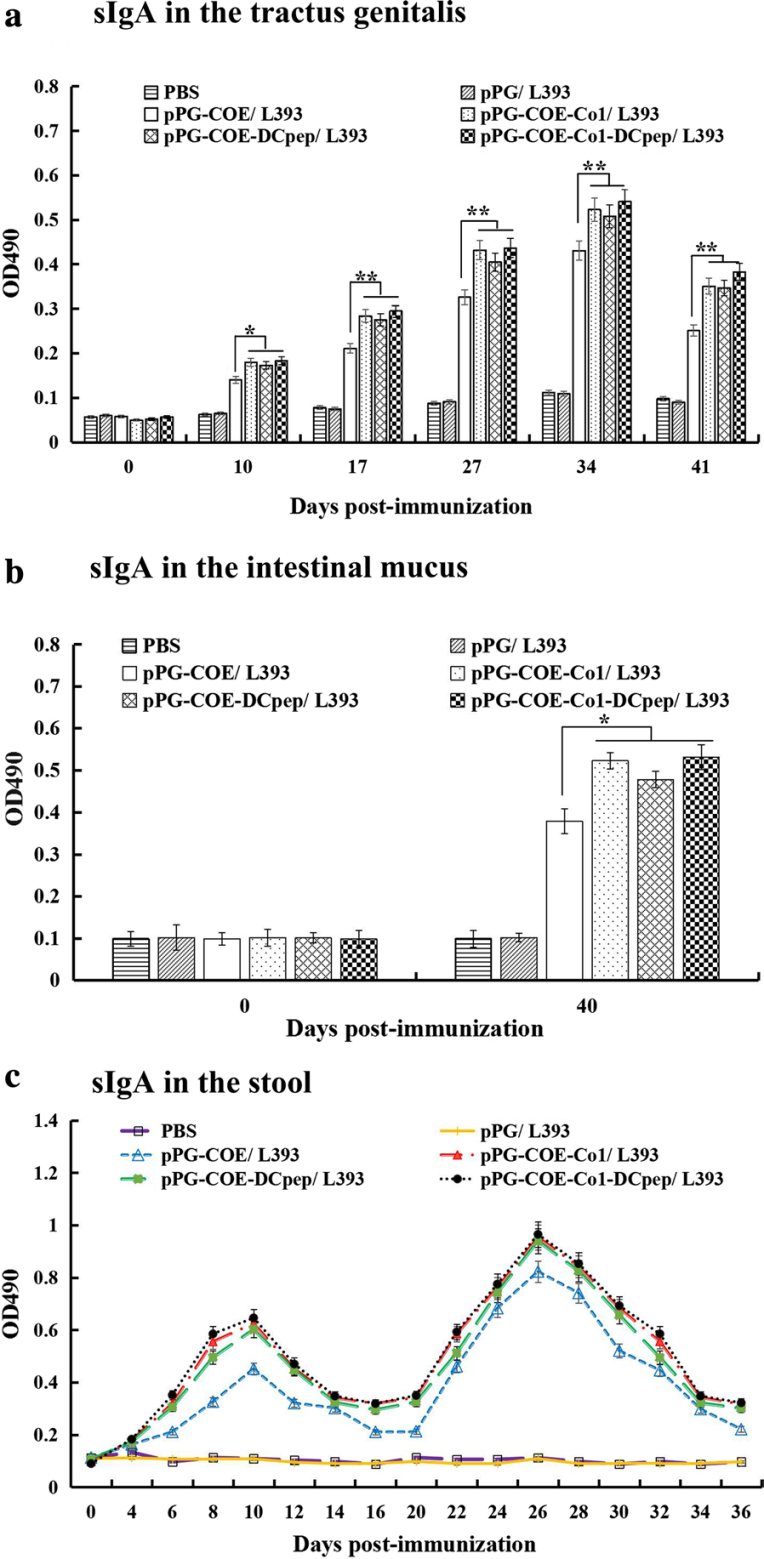
Anti-PEDV specific sIgA levels in the **a** external genital tract, **b** intestinal mucus and **c** stools post-immunization with recombinant strains. Bars represent the mean ± standard error value of each group (*p < 0.05, **p < 0.01 compared with pPG-COE/L393 group)

The immunogenicity of the recombinant *Lactobacillus* strains pPG-COE/L393, pPG-COE-Col/L393, pPG-COE-DCpep/L393, and pPG-COE-Col-DCpep/L393 in mice after oral immunization was evaluated; specifically, the mucosal and systemic immune responses were assessed by detecting the presence of anti-PEDV IgG and IgA antibodies by ELISA, respectively. The scheme of oral immunization and specimen collection is shown as Fig. 4. Our results showed significantly higher levels of antigen-specific systemic IgG antibodies (Fig. 5) on day 10 (*p* < 0.05) or on days 17, 27, and 41 (*p* < 0.01),and antigen-specific mucosal sIgA antibodies in the tractus genitalis (Fig. 6a) on day 10 (*p* < 0.05), or on days 17, 27, 34, and 41 (*p* < 0.01) post-immunization in mice orally administered with the recombinant strains pPG-COE-Col/L393, pPG-COE-DCpep/L393, and pPG-COE-Col-DCpep/L393 compared with the pPG-COE/L393 group. The same comparative results were observed with sIgA antibodies in the intestinal mucus on day 40 post-immunization (Fig. 6b). Figure 6c shows the trend of sIgA levels in the stools. After primary immunization, the sIgA antibody level rose rapidly to a peak on day 10, and subsequently declined until day 16. After the booster immunization, the sIgA antibody level increased rapidly and reached a peak on day 26, and the peak value was significantly higher than that of the first immunization. The sIgA antibody induced by recombinant strains with the targeting ligand groups increased faster than that induced in the pPG-COE/L393 group from the sixth day in the stools. However, the differences among the COE-Col, COE-DCpep, and COE-Col-DCpep groups in IgG or sIgA were not significant (*p* > 0.05).

### Proliferation of lymphocytes and cytokine ELISA

**Fig. 7:**
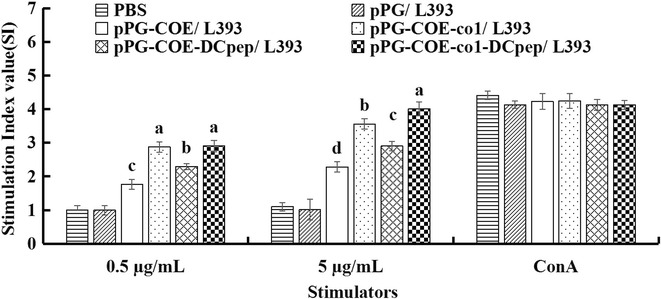
Lymphocyte proliferation, determined by MTT assay, in response to recombinant COE protein and concanavalin A as stimulating agents in immunized mice. Bars represent the mean ± standard error value of each group. Different letters indicate significant differences (*p* < 0.05)

The proliferation of splenocytes upon stimulation with COE protein was analyzed by MTT assay. Results showed that with an increase in the concentration of purified COE protein, the proliferation of splenocyte in mice followed the trend shown in Fig. 7: pPG-COE-Col-DCpep/L393 > pPG-COE-Col/L393 > pPG-COE-DCpep/L393 > pPG-COE/L393 (*p* < 0.05).

**Fig. 8:**
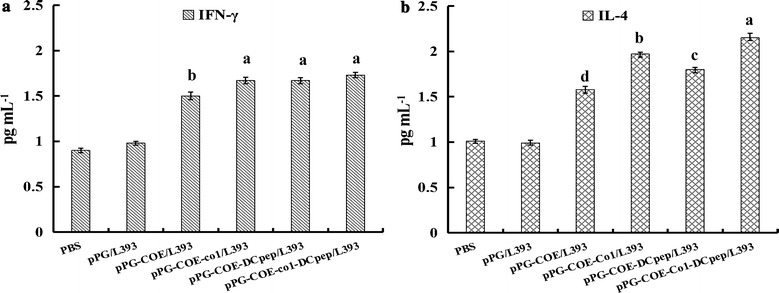
Cytokine levels of spleen cells from immunized mice. The purified core neutralizing epitope (COE) was added into cultured spleen cells as a stimulator, and **a** gamma interferon (IFN-γ) and **b** interleukin-4 (IL-4) were then detected. Different letters indicate significant differences (*p* < 0.05) and the same letters indicate no significant difference (*p* > 0.05)

Cytokine ELISA with culture supernatants harvested at 72 h showed that in response to COE, splenocytes from mice orally administered with the recombinant *L. casei* expressing COE-Col-DCpep, COE-Col, and COE-DCpep produced higher levels of the Th1-associated cytokine IFN-γ (Fig. 8a) and the Th2-associated cytokine IL-4 (Fig. 8b) compared to those in mice administered with recombinant *L. casei* expressing COE. As with the splenocyte proliferation, the same trend was observed with IL-4 levels: pPG-COE-Col-DCpep/L393 > pPG-COE-Col/L393 > pPG-COE-DCpep/L393 > pPG-COE/L393 (*p* < 0.05).

### PEDV-neutralizing activity

The neutralizing activity of sera from mice orally administered with the recombinant *L. casei* was evaluated by diluting antibodies and mixing with a maintained dose of the virus. Neutralization titer was calculated by the Reed–Muench statistical method. The neutralization titer of PBS and pPG/L393 groups was less than 1:2. The titers for PPG-COE/L393 (1:6), pPG-COE-Col/L393 (1:24), pPG-COE-DCpep/L393 (1:24), pPG-COE-Col-DCpep/L393 (1:36) indicated that mice administered with *L. casei* expressing COE-Col-DCpep possessed a stronger ability to neutralize the PEDV than ones administered with *L. casei* expressing COE-Col or COE-DCpep. The neutralizing activity of sera from COE-Col and COE-DCpep groups are similar. Recombinant strains with targeting ligand groups conferred a higher level of neutralization on PEDV infection compared with pPG-COE/L393. Moreover, no neutralizing activity was observed in the sera of mice administered with pPG/L393 or PBS.

## Discussion

Currently, PEDV has caused significant economic losses because of high mortality and loss of productivity. Subsequently, attenuated and inactivated PEDV vaccines have been developed [28, 29]. For reasons unknown, some vaccines based on classical PEDV strains have failed to control the more recent virulent PEDV strains in Asia [30]. The evolution of developing effective vaccines against PEDV has never ceased due to the presence of epidemic and variant strains. The S protein of coronaviruses is reported to be responsible for binding to cellular receptors in the early steps of infection and induces neutralizing antibodies in vivo [31]. Immunogens based on local virus isolates may help to prevent and control the outbreaks in this region. In this study, the gene homology of COE (499–638 amino acids in S protein) between classical PEDV strains and HLJ-2012 strain was 93.6–98.8%, and the COE cloned in this study did not show any insertion or deletion in its gene sequence.

Parenteral vaccination generally fails to induce mucosal immunity where PEDV infection is localized. Oral vaccination can stimulate a protective mucosal immune response as well as be crucial for passive lactogenic immunity [28]. A recent study suggested that successful generation of PEDV neutralizing antibodies in milk depends on oral immunization or oral exposure of sows, and that serum IgG antibody may not be a correlative immunity for PEDV [32]. IgG was dominant in the colostrum, but was decreased in sow milk with time. SIgA accounts for 40% of the total milk antibodies [33]. In order to deliver COE to the gastrointestinal tract by oral administration, genetically engineered lactobacillus constitutively surface-displaying COE was constructed as a particulate antigen for induction of immune responses and immunocyte memory. *L. casei* 393 can survive in bile salts or low pH in the gastrointestinal tract as well as colonize the intestines of mice and swine for a few days [34] to enhance the systemic and mucosal immune responses for orally administered antigens [35]. Constitutive expression without an inducer is generally preferred for in situ delivery of antigens by lactobacillus in consideration of productive stability and the high cost of inducers.

One of the essential considerations for oral vaccines is that the immunogen needs to penetrate the gut wall where epithelial cells are lined tightly, to be taken up by antigen presenting cells, which influence the immune efficiency. Antigen sampling occurs by transcytosis in the membrane of M cells, as well as in the dendrite extensions of dendritic cells into the lumen [36]. In view of this, *L. casei* 393 strains that expressed mammalian DCpep and Col fused separately or together with the immunogen for targeted vaccination were evaluated for their immune efficiency in mice. Theoretically, recombinant lactobacillus first adheres to the intestinal mucus competing for binding epitopes with pathogenic bacteria. Simultaneously, the recombinant *Lactobacillus* was guided by Col binding to the receptor of M cells to be imported into the dome area of the gut associated lymphoid tissue where DCs are present. Then DCpep allows COE to be captured by DCs by binding to its receptors. Moreover, COE fused with DCpep can be directly engulfed by DCs through their dendrites between the epithelial cells. Protective immunity can also be elicited by activated B cells secreting sIgA [37, 38] and activated T cells differentiation. DCs can present processed COE to T and B cells directly or migrate into the mesenteric lymph nodes for antigen presentation. DC-activated T and B cells can migrate to the periphery to induce specific immunity against the pathogen challenge [17]. Our data showed that recombinant strains with targeting ligands, which likely recruited DCs and M cells, promoted more rapid and stronger immune responses at the mucosal and systemic levels, compared with those induced by pPG-COE/*L*393.

In this study, COE-Col, COE-DCpep, and COE-Col-DCpep groups showed higher sIgA levels in the feces compared with the COE group, from the sixth day post immunization, and higher IgG and sIgA in the tractus genitalis from day 10 post immunization, which illustrates that in the early stages, targeting ligands can help elicit better immunogenicity more rapidly, especially sIgA levels, indicating the significance of our oral vaccine. To evaluate the effect on cellular immunity, splenocyte proliferation and cytokine levels were determined. Differential effects on specific cell-mediated immunity depend on the adjuvant dosage, *Lactobacillus* strains, and antigen used [39, 40]. Higher levels of Th2-associated IL-4 and Th1-associated IFN-γ in vaccine groups than those in the control group illustrated cellular immune response was induced. The trend of splenocyte proliferation and IL-4 levels indicate that targeting ligands may enhance the production of memory lymphocytes and humoral immunity (COE-Col-DCpep group > COE-Col group > COE-DCpep group > COE group > PBS group, *p* < 0.05). Other relevant researches also demonstrate that targeting peptides can enhance antigen-specific IgG, IgA, T cell responses [19, 21]. Of these, it is reported that the antibody levels from mice orally administrated *Lactobacillus* expressing antigen-DCpep, were comparable with that from mice in the group vaccinated with antigen-adjuvant (single subcutaneous injection) [19]. Another research showed that the Co1 ligand did not induce oral tolerance and the use of Co1 resulted in a skewed Th2-type immune response [21]. In addition, we evaluated neutralizing antibodies, which can neutralize the viral pathogen by binding to epitopes on antigens associated with virus neutralization. After neutralization, some parts of the virus’s infection cycle were inhibited, including cellular surface binding, fusion, entry, endocytosis, and replication [41]. Neutralizing antibodies have provided a quality correlate of vaccine efficacy for many licensed vaccines [42]. Assessment of PEDV-neutralizing activity illustrated that the COE-Col-DCpep group has the best neutralization potential suggesting that pPG-COE-Col-DCpep/L393 may be a promising vaccine candidate against PEDV infections.

The mucosal immune system properly balances pathogen surveillance and tolerance to dietary antigens and commensal microbes. And this indicates that mucosal antigens are generally less immunogenic than antigens delivered by other route. Potent adjuvants and delivery platforms are required for effective mucosal vaccination [43, 44]. It is reported that DCpep and Co1 would reduce the need for adjuvants to enhance immune responses and have mucosal adjuvant ability [9, 18, 21]. In this study, *L. casei* 393, which can colonize the intestine transiently, was applied as delivery vector and potent adjuvant themselves. Moreover, targeting ligands can favorably increase the bioavailability of the vaccine and help to elicit mucosal and systemic immune responses.

Further studies should focus on swine zoopery to investigate immune efficacy and protection. Although the BALB/c mouse is not a susceptible animal model for PEDV, our results to some extent indicate that surface-displaying COE-Col-DCpep *L. casei* could serve as a novel mucosal vaccine that provides opportunities for PEDV vaccine development.

## Conclusions

We used *L. casei* 393 as an antigen carrier to deliver COE fused with M cell and DC targeting ligands as an oral vaccine. Our results showed that pPG-COE-Col-DCpep/L393, pPG-COE-Col/L393, pPG-COE-DCpep/L393, were able to effectively induce immune responses at both the mucosal and systemic levels compared with pPG-COE/L393. Moreover, we found that *L. casei* 393 delivering the COE antigens in combination with DCpep and Col can promote stronger, more rapid antigen-specific immune responses in mice, suggesting it as a promising strategy for vaccine development against PEDV infection.

## Additional file

**Additional file 1.** The protein structure and sequence of fused COE-Col-DCpep predicted by SWISS-MODEL. Different colors in the sequence correspond to the structure.
