# Supplementary figures and images for: Oral recombinant Lactobacillus vaccine targeting the intestinal microfold cells and dendritic cells for delivering the core neutralizing epitope of porcine epidemic diarrhea virus
Source: Microb Cell Fact. 2018 Feb 9;17:20. doi: 10.1186/s12934-018-0861-7 (PMC5807822; doi:10.1186/s12934-018-0861-7)

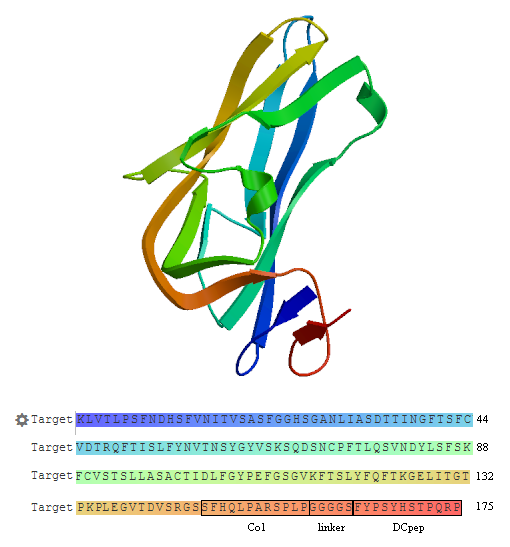

Supplement: Supplementary file 1 — Additional file 1. The protein structure and sequence of fused COE-Col-DCpep predicted by SWISS-MODEL. Different colors in the sequence correspond to the structure. [file 12934_2018_861_MOESM1_ESM.tif]
